# Organelle-specific localization of glutathione in plants grown under different light intensities and spectra

**DOI:** 10.1007/s00418-022-02103-2

**Published:** 2022-04-29

**Authors:** Anna Gasperl, Günther Zellnig, Gábor Kocsy, Maria Müller

**Affiliations:** 1 Institute of Biology, Plant Sciences, NAWI Graz, University of Graz, 8010 Graz, Austria; 2 Agricultural Institute, Centre for Agricultural Research, Eötvös Loránd Research Network, 2462 Martonvásár, Hungary

**Keywords:** Light intensity and spectrum, Subcellular glutathione content in plants, Oxidative stress, Redox state, Immunolabeling, roGFP

## Abstract

Plant ascorbate and glutathione metabolism counteracts oxidative stress mediated, for example, by excess light. In this review, we discuss the properties of immunocytochemistry and transmission electron microscopy, redox-sensitive dyes or probes and bright-field microscopy, confocal microscopy or fluorescence microscopy for the visualization and quantification of glutathione at the cellular or subcellular level in plants and the quantification of glutathione from isolated organelles. In previous studies, we showed that subcellular ascorbate and glutathione levels in *Arabidopsis* are affected by high light stress. The use of light-emitting diodes (LEDs) is gaining increasing importance in growing indoor crops and ornamental plants. A combination of different LED types allows custom-made combinations of wavelengths and prevents damage related to high photon flux rates. In this review we provide an overview on how different light spectra and light intensities affect glutathione metabolism at the cellular and subcellular levels in plants. Findings obtained in our most recent study demonstrate that both light intensity and spectrum significantly affected glutathione metabolism in wheat at the transcriptional level and caused genotype-specific reactions in the investigated *Arabidopsis* lines.

## Introduction

The increasing number of sudden drought, flooding or late frost events is challenging for current and future human food supply. Maize, rice and wheat are the most important staple foods worldwide (Pariona 2019). In 2019, 15% of the world’s wheat production areas did not receive enough irrigation water (Trnka et al. 2019). By the end of the twenty-first century, these authors extrapolated a high risk for simultaneous water shortages in up to 60% of the world’s wheat production areas, with the degree of risk dependent on mankind’s efforts to alleviate climate change.

Wheat, the model plant *Arabidopsis thaliana* and other C_3_ photosynthesis-type plants of temperate origin are highly susceptible to drought, which causes substantial yield losses in these plants due to the effects of osmotic and oxidative stress (Gill and Tuteja 2010). Plant organelles are differentially affected by oxidative stress. Excess levels of reactive oxygen species (ROS) as a consequence of intense and/or persistent abiotic or biotic stress frequently occur in the vicinity of electron transport chains. Consequently, in plants, thylakoid membranes in chloroplasts (Noctor et al. 2018) and the mitochondrial inner membranes (Huang et al. 2016), which are involved in intermediate steps of photosynthesis and respiration, respectively, are particularly prone to oxidative damage. At the cellular and subcellular levels, persistent oxidative stress damages RNA, DNA, pigments and proteins and causes the disintegration of membranes through lipid peroxidation, which eventually leads to de-compartmentation and cell death (Gill and Tuteja 2010; Karuppanapandian et al. 2011; Dorion et al. 2021). The oxidative stress response in plants is therefore also cell compartment specific (Zechmann 2014, 2020). In this context, it is highly important to gain a better understanding of the complex and finely tuned regulatory and signaling networks functioning in plant stress response at the subcellular level.

 The redox state in plants results from a combined effect of environmental conditions and genetic regulation. Plants can adapt their growth and development to mild environmental changes and/or seasonal or annual alterations, whereas sudden and/or strong environmental changes over an extended period of time may lead to tissue damage and death (Kocsy et al. 2013). The growth and development of plants is driven by photosynthesis, which is most efficient within the blue and red spectrum of light. During photosynthesis, electrons are transferred from water via an electron transfer chain to nicotinamide adenine dinucleotide phosphate (NADP^+^), using energy absorbed from sunlight. The electrochemical gradient between the thylakoid lumen and the stroma initiates a proton gradient that facilitates the phosphorylation of adenosine diphosphate (ADP). The products of this phosphorylation, adenosine triphosphate (ATP) and reduced NADP^+^ (NADPH), are used for carbon fixation and the incorporation of carbon into organic compounds. The accumulation of ROS depends on a functional redox-homeostasis in the chloroplast and other cell compartments. For example, superoxide radicals (O_2_^−^) are generated by the Mehler reaction from oxygen and two O_2_^−^ radicals, and are subsequently dismutated by superoxide dismutase (SOD; EC 1.15.1.1) to hydrogen peroxide (H_2_O_2_) and oxygen. If the light energy absorbed exceeds the scavenging capacity for ROS, irreversible damage from singlet oxygen (^1^O_2_) in photosystem II (PS II) results in photoinhibition. Carotenoids are involved in quenching ^1^O_2_ and xanthophyll carotenoids facilitate the dissipation of excess absorbed light energy as heat (non-photochemical quenching). H_2_O_2_ and other ROS detoxification in chloroplasts is mediated by the ascorbate–glutathione (or Foyer-Halliwell-Asada) cycle or by redox transmitters (peroxiredoxins).

 Due to their sessile nature, plants are required to develop a fast and versatile response system to fluctuating and/or excess light intensity (Scheibe and Dietz 2012; Ding et al. 2016; Mullineaux et al. 2018; Turkan et al. 2018). Interestingly, in one study, stress signaling transcripts strongly reacted to a single excess light trigger at 1000 µmol m^−2^ s^−1^ photon flux density in non-primed *Arabidopsis* plants, while the same transcripts in primed plants (repeatedly pre-treated with excess light at 1000 µmol m^−2^ s^−1^) remained largely unaffected. Ganguly et al. (2019) concluded that primed *Arabidopsis* plants were more tolerant to excess light compared to non-primed plants, due to adjustments in the efficiency of photosynthetic electron transport and because priming was independent from functioning DNA methylation. Sudden fluctuations between full sunlight and shade interferes with the excitation state (and thus redox-homoeostasis) of the two photosystems and causes ROS accumulation along the electron transport chain. Plants are able to re-arrange the antenna structure of the photosystems (state transition) in chloroplasts within minutes. Given that chlorophyll and carotenoids primarily absorb in the red spectrum, but not in the far-red light spectrum, understory plants or leaves of lower nodes on a plant need long-term adaptation strategies for efficient electron transport during photosynthesis. Long-term (hours to days) response to low red/far-red (shade) conditions involves adaptations in photosystem stoichiometry (Dietzel et al. 2008). In *Phaseolus vulgaris* plants cultivated under a low red/far-red ratio (0.2 instead of the normal 1.1), which resembles light availability to ‘shade’ leaves, leaf ascorbate (Asc) and glutathione contents, the activity of antioxidant enzymes and respiration rates indeed were lower compared to the control white light regime (Bartoli et al. 2009). Excess ROS may, however, be formed in seedlings grown under continuous far-red light because the reduction of protochlorophyllide to chlorophyll is impaired by the limited ability of protochlorophyllide to absorb far-red light and by the transcriptional repression (mediated by phytochrome A [phyA]) of the oxidoreductase involved (Runge et al. 1996; Sheerin and Hiltbrunner 2017).

Light is also involved in the signal transduction of morphogenesis (plant elongation, leaf expansion), stomatal opening, the circadian clock and flowering, all of which are sensed by wavelength-specific photoreceptors. The following photreceptors have been identified in plants: for the UV/A-blue wavelengths, the cryptochromes (cry), phototropins and members of the Zeitlupe family; for the red and far-red wavelengths, phytochromes; and for UV/B, UV RESISTANCE LOCUS8, a UV-B photoreceptor, which also reacts to short wavelength UV/A (Rai et al. 2021; Kami et al. 2010; Chen et al. 2004). Far-red light is sensed by phyA and is involved in the regulation of plant development and growth by inducing phyA or repressing phytochrome B, whereas changes in the ratio of red/far-red light are primarily sensed by phytochrome B, which mediates shade response or ‘shade avoidance syndrome’ (Sheerin and Hiltbrunner 2017; Viczián et al. 2017). Phytochromes and cryptochromes are associated with phytochrome-interacting factors that mediate downstream gene regulation (Kianianmomeni 2014; Pedmale et al. 2016; Pham et al. 2018). The subcellular localization of photoreceptors is light dependent. While most of the *Arabidopsis* phytochromes have been localized in the cytosol in the dark, under light conditons they are translocated into the nucleus. One exception is cry1, which is localized in the nucleus in the dark, but mainly in the cytosol in the light (Chen et al. 2004) and activated by light-dependent changes in the redox state of a cofactor (flavin) (Bouly et al. 2007).

### Prevention of oxidative damage in plant organelles during osmotic stress

On hot, dry days C_3_-photosynthesis-type plants like wheat, rice or *Arabiopsis* close their stomata to prevent water loss. Under sunny conditions, photons are highly abundant, but closing the stomata limits gas exchange and hence the availability of the electron acceptors ADP^+^ and NADP^+^ from the Calvin–Benson cycle. To prevent thylakoid electron transport chain overload, photorespiration is induced, which on the one hand supplies ADP^+^ and NADP^+^ and on the other hand regenerates 3-phosphoglycerate from 2-phosphoglycolate. However, the photorespiratory cycle in peroxisomes, which allows the regeneration of glycolate from photorespiration for primary metabolism, produces H_2_O_2_, which serves a signaling function at low concentrations, but is toxic at high concentrations and is balanced via Asc, glutathione and the enzyme catalase (CAT; EC 1.11.1.6). In mitochondria, ROS, in particular O_2_^−^, accumulate under conditions of limited ADP. However, under excess light, the respiratory rate increases, which in turn aids photochemical quenching, and an alternative oxidase isoform (AOX) is expressed, which limits the energy efficiency, thereby avoiding intermediate ROS formation (Gill and Tuteja 2010; Exposito-Rodriguez et al. 2017; Mullineaux et al. 2018).

 Plants are able to counteract oxidative stress, with redox changes triggering and modifying physiological processes. ROS, reactive nitrogen and reactive sulfur species together with antioxidants play a pivotal regulatory role in these adaptation and defense mechanisms at the transcriptome, proteome and metabolome levels. Asc and glutathione are the most important antioxidants in plants, given their functions as coenzyme and posttranslational modifier, respectively (Foyer and Noctor 2005; Noctor et al. 2012; Kocsy et al. 2013; Olson 2020). In plants, the concerted action of Asc, glutathione and other non-proteinaceous (NADP^+^/H) and proteinaceous (ascorbate peroxidase [APX], EC 1.11.1.11; monodehydroascorbate reductase [EC 1.6.5.4]; dehydroascorbate reductase [EC 1.8.5.1; glutathione reductase [EC 1.8.1.7]) components of the ascorbate–glutathione cycle (or Foyer-Halliwell-Asada cycle) allows the detoxification of excess H_2_O_2_ (Foyer and Halliwell 1976; Asada 1999; Foyer and Noctor 2011). The pool of reduced glutathione (GSH) is fueled by glutathione reductase (GR) activity, which catalyzes the reduction of glutathione disulfide (GSSG) to two molecules of GSH by electron transfer from NADPH. Compartment-specific GRs are found in the cytosol, chloroplasts, mitochondria and peroxisomes (reviewed by Csiszár et al. 2016). *Arabidopsis* GR2, located in the chloroplast and mitochondria, helps to maintain the function of PS II under excess light conditions by increasing the level of glutathione (Ding et al. 2016). The lack of reducing power in GR2 null mutants can be partially complemented by the ATP-binding cassette transporter and thioredoxin system in mitochondria, but not in chloroplasts (Marty et al. 2019). Similarly, GR1 knockout lines of the model moss *Physcomitrella patens* are not able to maintain a full reducing environment in the chloroplast stroma under excess light (Müller-Schüssele et al. 2020). An increase in the amount and ratio of GSSG and GSH with increasing light intensity has been reported for *Arabidopsis* (Heyneke et al. 2013; Choudhury et al. 2018; Gasperl et al. 2021) and wheat (Gasperl et al. 2021). Excess light at 500 µmol m^−2^ s^−1^ was found to induce cysteine and glutathione synthesis and glutathione reduction in wheat at the transcriptional level (Monostori et al. 2018; Gyugos et al. 2019; Toldi et al. 2019; Gasperl et al. 2021). Glutathione synthesis is ATP-consuming and depends on the availability of the amino acids cysteine, glutamine and glycine and requires two subsequent reactions, in which γ-glutamylcysteine synthetase (γ-ECS or GSH1, EC 6.3.2.2) first catalyzes the formation of γ-glutamylcysteine (γ-EC) from cysteine and glutamine and glutathione synthetase (GSHS or GSHS2, EC 6.3.2.3), then catalyzes the formation of glutathione from γ-EC and glycine. While the first step of glutathione synthesis is restricted to chloroplasts in *Arabidopsis* and wheat, the second step is primarily confined to the cytosol (Noctor et al. 2012). Koffler et al. (2011) found glutathione synthesis in *Arabidopsis* to be limited to the availability of γ-EC in chloroplasts and the cytosol. In a drought-tolerant wheat variety (Plainsman), the recovery photosynthesis rate and recovery growth rate improved with increasing light intensity, which may have partly been due to a higher cysteine availability for sufficient glutathione synthesis after drought stress, evident from the increase in cysteine and glutathione levels and expression of the glutathione synthetase gene *GSHS2* (Gyugos et al. 2019). Interestingly, shade pre-treated (low red/far-red light ratio of 0.4 ~ 0.6) soybean seedlings likewise coped better with drought by an improved ROS scavenging system (Asghar et al. 2020). Glutathione further plays an important role in the detoxification of xeno- or endobiotics, where glutathione transferases (GTs, EC 2.5.1.18) catalyze the conjugation of glutathione to electrophilic compounds, which facilitates transport into the vacuole for metabolization (Labrou et al. 2015). GTs in plants are grouped into the cytosolic, mitochondrial and microsomal super families. Certain types of GTs have antioxidant properties, such as dehydroascorbate reductase or glutathione peroxidases, and others are involved in hormone signaling (Noctor et al. 2012; Csiszár et al. 2016). Darkness, low light or shade mostly reduce the activity of GTs, while high light intensity elevates the activity and gene expression of GTs (Gallé et al. 2018).

### Subcellular detoxification model for excess H_2_O_2_ in* Arabidopsis* under excess light or lower red/far-red ratio and regulation of antioxidants by light intensity and spectrum composition

**Fig. 1:**
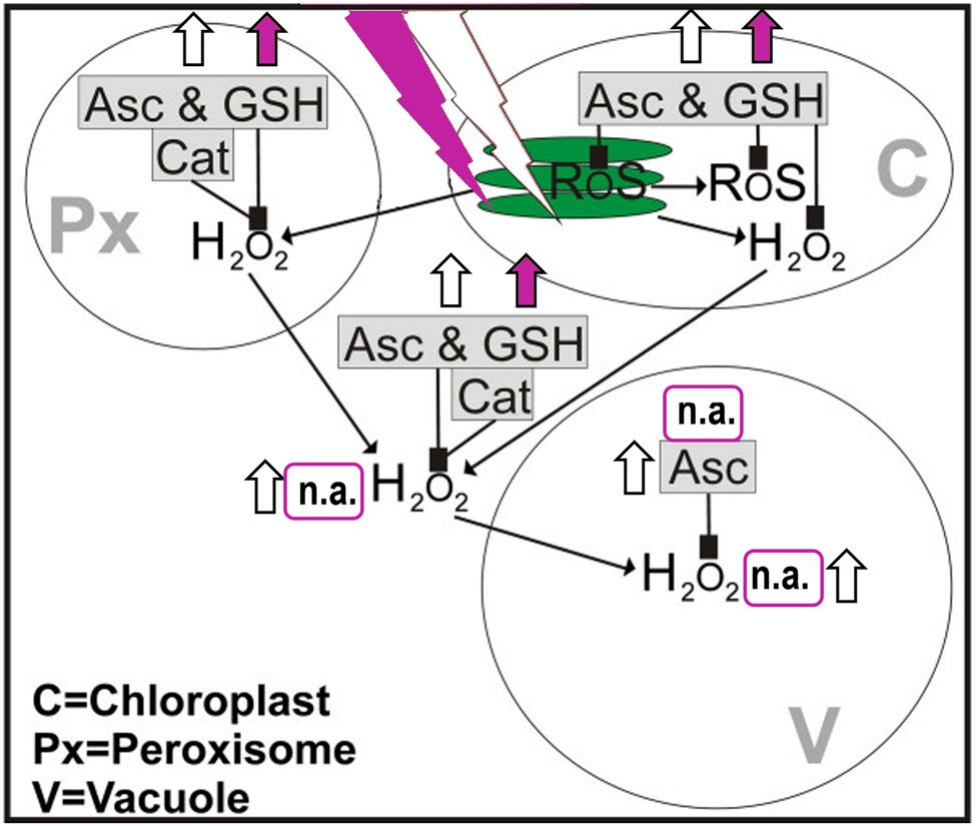
Adapted model of subcellular reactive oxygen species (ROS) accumulation and detoxification by antioxidants and catalase in plants under conditions of excess light or white light with lower red/far-red ratio (graph reproduced using Corel® Photo Paint 2019 [Corel Corp., Ottawa, ON, Canada] with modifications from Heyneke et al. 2013). Line drawing proposing a model of the effects of high light stress or of a decreased red/far-red ratio on the subcellular accumulation of ROS in *Arabidopsis thaliana* and wheat with special focus on the compartment-specific detoxification of hydrogen peroxide (H_2_O_2_) by ascorbate (Asc), catalase (Cat) and reduced glutathione (GSH). Excess light stress (indicated by white thunderbolt) induces the generation of ROS and H_2_O_2_ in chloroplasts (C) and in peroxisomes (Px) by overstraining the electron transport chain in thylakoids (green ovals inside the chloroplast) and through photorespiration, respectively. Asc, Cat and GSH detoxify and suppress the accumulation of ROS and H_2_O_2_ in these cell compartments. Accumulation of H_2_O_2_ (detected by cerium chloride) and subcellular antioxidants (detected via immunolabeling) by excess light in *Arabidopsis* and wheat is indicated by a white upwards arrow. White light with decreased red/far-red ratio (indicated by pink thunderbolt) induces the generation of ROS and H_2_O_2_ in chloroplasts of seedlings compared to seedlings grown in white light with normal red/far-red ratio by inefficient chlorophyll biosynthesis (impaired reduction of protochlorophyllide to chlorophyllide by protochlorophyllide oxidoreductase [POR] activity). With increasing stress, including high light intensities or shade (limited reducing power and assimilates), H_2_O_2_ leaks from chloroplasts and peroxisomes into the cytosol and eventually into vacuoles. In shaded leaves (lower red/far-red ratio), an imbalance in the excitation of photosystems II and I induces ROS and H_2_O_2_ formation in chloroplasts. Accumulation of subcellular glutathione (detected via immunolabeling) by a decreased red/far-red ratio in the *Arabidopsis pad2-1* mutant and wheat is indicated by a pink upwards arrow. *Arabidopsis* wild-type and *vtc2-1* mutant cell compartments were largely unaffected by a decreased red/far-red ratio. Whereas Asc, Cat and GSH detoxify H_2_O_2_ also in the cytosol, only Asc is involved in the detoxification of H_2_O_2_ in vacuoles (V), where it helps to reduce phenoxyl radicals created by oxidation of phenols by H_2_O_2_. H_2_O_2_ was detected after short-term (4 h) exposure to high light stress, but was successfully detoxified in the long term (2 weeks). H_2_O_2_ and Asc were not analyzed (n.a.) under a decreased red/far-red ratio (Runge et al. 1996; Takahama 2004; Dietzel et al. 2008; Scheibe and Dietz 2012; Kim and Apel 2013; Sheerin and Hiltbrunner 2017; Gasperl et al. 2021)

Increased H_2_O_2_ formation was sensed in chloroplast stroma, the cytosol and nuclei of genetically modified tobacco HyPer2 (fluorescent H_2_O_2_ biosensor) plants cultivated in excess light at 1000 µmol m^−2^ s^−1^ for 1 h. Given that chloroplast-derived H_2_O_2_ is detected in nuclei, chloroplast-derived H_2_O_2_ may facilitate a fast response to changes in light intensity at the gene expression level (Exposito-Rodriguez et al. 2017). It was proposed that excess H_2_O_2_ from overstrained electron transport chains in *Arabidopsis* chloroplasts and from photorespiration in peroxisomes may leak into the cytosol and vacuole with increasing light intensity. Excess H_2_O_2_ can be detoxified by Asc, GSH, CAT and APX activity in peroxisomes and the cytosol, by Asc, GSH and APX activity in chloroplasts, while in vacuoles only Asc is available as a reductant and guaiacol-type peroxidase (EC 1.11.1.7) activity aids in scavenging excess H_2_O_2_ (Fig. 1) (Takahama 2004; Heyneke et al. 2013; Zipor and Oren-Shamir 2013; Gasperl et al. 2021).

Indeed, short-term (4 h) excess light at 1500 µmol m^−2^ s^−1^ led to the accumulation of H_2_O_2_ in the cytosol and along the tonoplast, but was absent in cell compartments after 2 weeks of excess light treatment (Heyneke et al. 2013). This detoxification model for excess ROS and H_2_O_2_ in *Arabidopsis* is based on findings derived from high light intensity treatments using compact fluorescent lamps (Plug and Grow, 6400 K, white/blue spectrum; Agriculture Trading AG, Walenstadt, Switzerland), at 1500 µmol m^−2^ s^−1^ for 4 h, which resulted in the accumulation of H_2_O_2_ in the cytosol and along the tonoplast in vacuoles, increased density of immunolabeled Asc and total glutathione (= sum of GSH and GSSG) in chloroplasts, peroxisomes and the cytosol, degradation of thylakoid membranes (plastoglobuli formation) and an overall increase in H_2_O_2_ level and CAT activity in the leaves after 14 days of treatment. Interestingly, the *Arabidopsis* lines showed a genotype-specific adaptation to 14 days of excess light. The glutathione-deficient *Arabidopsis* mutant *pad2-1* ( seemed to compensate its lower level of total glutathione by accumulating more Asc in peroxisomes, whereas the opposite was true for the Asc-deficient *Arabidopsis* mutant *vtc2-1*, which showed an increase in chloroplast and nuclei total glutathione content (Heyneke et al. 2013). In our most recent studies (Monostori et al. 2018; Toldi et al. 2019; Gasperl et al. 2021), we used continuous wide-spectrum light-emitting diodes (LEDs) to manipulate the light intensity (Lumileds LXZ-5790y; Philips, Amsterdam, The Netherlands) and spectrum composition (narrow-spectrum LEDs with dominant wavelengths of 448 nm [Lumileds LXZ1-PR01; Philips], 665 nm [Lumileds LXZ1-PA01; Philips] and 750 nm [2ER101FX0000001; Edison Edixeon, Moers, Germany). Under white light with lower red/far-red ratio, we propose an increase in ROS and H_2_O_2_ in chloroplasts followed by an increase in these molecules in other subcellular compartments due to inefficient chlorophyll biosynthesis in seedlings after the transition to white light. In mature shaded leaves, an imbalance in the excitation states of the two photosystems may increase ROS and H_2_O_2_ in chloroplasts. With increasing stress from a limited supply of reducing power (NADPH, ATP) and assimilates, H_2_O_2_ may subsequently accumulate in other cell compartments. These compartment-specific redox changes induce the accumulation and redistribution of antioxidants and associated enzyme activities (see Fig. 1; Runge et al. 1996; Dietzel et al. 2008; Scheibe and Dietz 2012; Kim and Apel 2013; Sheerin and Hiltbrunner 2017; Gasperl et al. 2021). LEDs have gained more and more importance as energy-efficient plant growth light sources during the last years and have been used to study the involvement of light, with special focus on red and blue spectra, in the regulation of growth and development of the model plant *Arabidopsis thaliana* and various crop plants; in this context, the enhanced accumulation of specific metabolites is also of great interest (Li et al. 2017; Monostori et al. 2018; Kong and Zheng 2020; Jenkins 2021; Samuolienė et al. 2020; Appolloni et al. 2022).

## Effect of light intensity and spectrum on genes encoding compartment-specific enzymes of glutathione metabolism

Changes in light intensity or spectrum may influence the level of total glutathione and its reduction state (GSSG/GSH) through modification of the activity of the proteins and the expression of the genes associated with glutathione metabolism and transport in the individual organelles. Regarding glutathione synthesis, its first step, the formation of γ-EC takes place in the chloroplasts and its transcriptional regulation by light intensity (higher expression under conditions of low light intensity than under normal light intensity) and far-red light (lower expression under white light with decreased red/far-red ratio) was shown in wheat (Gasperl et al. 2021). The expression of the two genes encoding the enzyme of the second step of glutathione synthesis (GSHS) in the cytosol and chloroplast increased with increasing light intensity, and this change was accompanied by an increase in total glutathione contents in these cell compartments. The expression of the gene encoding the chloroplastic form of this enzyme was one magnitude lower than that of the gene of the cytoplasmic form. Among the genes associated with GSSG reduction, the expression of the gene of cytosolic GR was also increased under conditions of high light intensity, but not in far-red light conditions, in wheat (Gasperl et al. 2021). These observations indicate that light conditions may also control the glutathione-dependent subcellular redox environment at the transcriptional level.

The tissue- and cell compartment-specific enzyme activity and gene expression of GTs is also influenced by both light intensity and quality (Chen et al. 2007; Gallé et al. 2018). A tau class GT, the FIN219-interacting protein 1 (FIP1), which interacts with Far-Red Insensitive 219 (FIN219), is involved in the phytochrome A-mediated signaling in *Arabidopsis* (Chen et al. 2007). This process in turn regulates cell elongation and flowering in response to light. *FIP1*gene transcription was induced by far-red light and the FIP1 protein was localized in the nucleus and cytoplasm. Thus, light spectrum-dependent changes in its activity may influence glutathione content in these compartments. Another GT gene, *AtGSTU17*, also participates in the signaling process associated with phytochrome A and controls the size and redox state of the glutathione pool in *Arabidopsis*, which in turn affect development (Jiang et al. 2010). The effect of blue and red light on the expression of GT and other genes encoding antioxidant enzymes was also shown in leaf tissues of maize seedlings (Liu and Zhang 2021). Glutathione contents and GR activities increased after long-term exposure to a combination of continuous red and blue light in lettuce (Zha et al. 2019). Unfortunately, in the latter studies, the effect of spectrum on genes encoding the isozymes in various organelles was not investigated, but their specific and different regulation can be assumed based on the observations in *Arabidopsis* and wheat (Chen et al. 2007; Gasperl et al. 2021).

## Quantification of glutathione in plant cell compartments

Methods for quantifying glutathione in plant cell compartments were concisely evaluated by Zechmann in 2014. Each of the approaches evaluated here has its own specific advantages and limitations, which are summarized in Table 1. Additional details on targets for redox-sensitive green fluorescence protein (roGFP) are included from Bratt et al. (2016), as are additional details on glutathione quantification from isolated organelles from Hajdinák et al. (2019) and Pradedova et al. (2019). Briefly, subcellular glutathione can be bound in situ or in vivo to glutathione-specific antibodies (total glutathione = sum of GSH and GSSG), redox-sensitive dyes (monobromobimane [mbrB] or monochlorobimane [mchlB]; GSH) or roGFP probes (GSH, GSSG), visualized by transmission electron microscopy (TEM), bright-field microscopy, confocal-laser scanning microscopy (CLSM) or fluorescence microscopy, respectively, and quantified by software-assisted image analysis. Total glutathione localization by use of a specific antibody (henceforth referred to as immunolabeling) on ultrathin sections (80 nm) of embedded samples preserves the current physiological state with respect to subcellular total glutathione distribution and labeling density in situ. Immunolabeling of total glutathione is possible in all plant cell compartments in which glutathione either plays a major role as an antioxidant (chloroplasts, the cytosol, mitochondria, peroxisomes, nuclei) and/or where glutathione is synthesized (chloroplasts, the cytosol) and/or reduced (chloroplasts, the cytosol, mitochondria, peroxisomes). Tolin et al. (2013) verified the accumulation of glutathione with this method in the apoplast of the *Arabidopsis ggt-1* mutant (GGT-1 knockout), which lacks glutathione degradation (by gamma-glutamyl transferase/transpeptidase [GGT-1]) in the apoplast. In the study of Queval et al. (2011), the accumulation of glutathione was detected in vacuoles of the catalase-deficient *Arabidopsis cat2* mutant. These results indicate that in *Arabidopsis* wildtype plants, glutathione levels are either not present or the level is too low to be detected with immunolabeling methods in vacuoles and the apoplast.

**Table 1:** Quantification of glutathione in plant cell compartments

Antibody/dye/probe	Cell compartments	Target	Visualization	Quantification	Advantage(s)	Limitations
GSH-specific antibody	mit, chl, per, nuc, vac, cw	Total glutathione	Transmission electron microscopy (TEM)	Software-assisted image analysis	- Indicates in situ situation - Deep cell layers accessible - High resolution (> 10 nm) - Local changes accessible - Small samples	- Fixation of samples required - Antibody availability/specificity
Monobromo-/monochlorobimane	cyt, nuc	GSH	Bright-field or fluorescence or confocal-laser scanning microscopy	Software-assisted image analysis	- Indicates *in-vivo* situation	- Only thin organs/tissues accessible - Microscope resolution (~ 200 nm) - Specificity of dye limited infiltration of compartments - Sample preparation/microscope properties: potential stress sources
Redox-sensitive GFP	mit, chl, cyt, ER, per	GSH, GSSG	Fluorescence or confocal-laser scanning microscopy	Software-assisted image analysis or microplate reader	- Indicates in vivo situation - Redox state of glutathione - Redox state of compartments	- Only thin organs/tissues accessible - Probe availability/specificity Sample preparation/microscope properties: potential stress sources requires genetical modification of target plant
Monobromo-/monochlorobimane	mit, chl, per, vac, apo, leu	GSH, GSSG	Not applicable	HPLC or spectrophotometer	- Discrimination between GSH and - GSSG redox state of compartments	- High amount of plant material washing out or redistribution of GSH, GSSG - In vivo situation unclear

Based on results reported by Zechmann (2014), Bratt et al. (2016), Hajdinák et al. (2019) and Pradedova et al. (2019)

*apo* Apoplast, *cyt* cytosol, *cw* cell wall,* ER* endoplasmic reticulum, *GFP* green fluorescent protein, *GSH* reduced glutathione, *GSSG* oxidized glutathione (glutathione disulfide), *HPLC* high-performance liquid chromatography, *leu* leucoplast,* mit *mitochondrium, *nuc* nucleus,* per* peroxisome, total glutathione sum of GSH and GSSG, *vac* vacuole

Further, deep cell layers, such as the mesophyll or vascular tissue, are accessible and the high resolution (~ 0.2 nm) of a TEM allows the visualization of even local, subcompartmental changes. The resolution limit for immunolabeling of total glutathione, however, is > 10 nm due to the size of the complex formed by glutathione, the primary (anti-glutathione rabbit polyclonal immunoglobulin G [IgG]) antibody and the secondary (goat anti-rabbit IgG) antibody which is conjugated to a gold particle of 10 nm in diameter (Zechmann et al. 2008a). Tissue samples of 1.5 mm^2^ are of sufficient size for study, thus saving plant material for additional analyses (such as studies on gene expression and metabolite composition). However, the immunolabeling approach requires careful sample fixation and is momentarily limited to a primary antibody which cannot distinguish between the GSH and GSSG. The use of redox-sensitive mbrB or mchlB and visualization of reduced glutathione via bright-field, fluorescence microscopy or CLSM, by contrast, allows insights into the in vivo situation within the nucleus and cytosol. Other plant cell compartments and the glutathione distribution in the mesophyll or vascular tissue are not accessible due to the limited infiltration potential of redox-sensitive dyes and/or a microscope resolution of approx. 200 nm. Sample preparation and microscope properties are potential stress sources and can thus influence glutathione redox state and distribution within the compartments. Cell compartment-specific, roGFPs, visualized via a fluorescence microscope or CLSM, indicate the in vivo redox state of glutathione in mitochondria, chloroplasts, the cytosol, peroxisomes (primarily GSH) and the endoplasmic reticulum (ER; primarily GSSG). Again, examinations are limited to thin organs/tissues and outer cell layers, and sample preparation or microscope properties are potential stress sources. Probes are mainly available for *Arabidopsis* and require genetical modifications of the target plant.

Alternatively, glutathione can be quantified after reduction of GSSG in the sample with dithiotreitol (DTT) and subsequent derivatization of GSH with mbrB or mchlB via a reverse-phase high-performance liquid chromatography (HPLC) system equipped with a fluorescent or UV detector, or spectrophotometrically after a GR-mediated glutathione recycling assay (total glutathione) from cell organelles after isolation or fractionation. To assess the GSSG fraction, first, thiol groups of GSH are blocked with N-ethylmaleimide (NEM) in a subsample, followed by removal of excess NEM with toluene prior to reduction of GSSG to GSH with DTT and derivatization via mbrB (Kranner 1998; Kranner and Grill 1993; Roach et al. 2018) or mchlB (Hajdinák et al. 2019). Similarly, NEM or vinylpyridine is added to the glutathione recycling assay reaction mix, and the GSH content can be calculated by the subtraction of GSSG from total glutathione. Hajdinák et al. (2019) found that the use of mchlB and HPLC analysis yielded more exact results compared to the glutathione recycling assay when applied to *Arabidopsis* suspension cell cultures, and mitochondrial, microsomal (ER) and cytosol fractions. HPLC with UV detection facilitates the quantification of GSH and GSSG without the need of derivatization. Such an approach has recently been applied to leucoplasts and vacuoles isolated from storage parenchyma cells of red beet (*Beta vulgaris* L.) (Pradedova et al. 2019). Generally, great care has to be taken during sample preparation to avoid auto-oxidation of GSH, washing out of and/or redistributing of glutathione between cell compartments. Such cross-contamination or additional stress to the sample may occur by applying too much pressure during tissue infiltration or due to microscope properties (strong light source, high temperature, lack of oxygen, water stress), but likewise during organelle isolation or fractionation (Zechmann 2014). According to Hajdinák et al. (2019), the addition of 1 mM mchlB to organelle isolation buffer is recommended to prevent auto-oxidation of GSH during the fractionation process.

Glutathione concentrations in individual cell compartments, calculated from a combination of data on the volume of cell compartments, with total glutathione immunolabeling density and glutathione concentrations in whole *Arabidopsis* leaf tissue (Queval et al. 2011; Han et al. 2013; Koffler et al. 2013; Zechmann 2020; Dorion et al. 2021) are in the millimolar range (mitochondria [15 mM], > nuclei [6.4 mM], > cytosol [4.5 mM], > peroxisomes [4.4 mM], > chloroplasts [1.2 mM], > vacuole [0.08 mM]). Similar glutathione concentrations have been found in earlier studies in animal tissue or *Arabidopsis* for mitochondria (Wahlländer et al. 1979; García-Ruiz et al. 1994; Meyer et al. 2001; Krueger et al. 2009), whereas the glutathione concentration was higher in animal cytosol (Wahlländer et al. 1979; García-Ruiz et al. 1994), and calculated cytosol levels were lower in young wheat leaves (Noctor et al. 2002). The reported chloroplast and vacuole glutathione concentration differs markedly among plant species (0.5–5 mM and 0.08–0.7 mM, respectively), which might be due to redistribution of glutathione via compartment-specific glutathione transporters (identified transporters summarized by Dorion et al. 2021) and/or sample handling during organelle isolation (discussed in detail by Noctor et al. 2002; Krueger et al. 2009; Hajdinák et al. 2019). Traces of 0.003 mM glutathione were detected in barley apoplast extract (Vanacker et al. 1998). Although this very low glutathione concentration in the apoplast may not be sufficient to act as antioxidant, such as during biotic stress (fungal infection), glutathione and/or its redox state may alternatively have a defense-signaling function in this cell compartment (Zechmann 2020). Given that the total glutathione concentration of different C_3_-type plants is highest in mitochondria (Zechmann et al. 2008a; Zechmann and Müller 2010; Heyneke et al. 2013; Koffler et al. 2013; Müller et al. 2014; Vidović et al. 2016; Gasperl et al. 2021) and increases with leaf age in *Arabidopsis* (Koffler et al. 2013), which is probably induced by higher ROS accumulation in older leaves, this high and comparatively stable glutathione level seems to be crucial for mitochondria and cell functioning and viability under non-stress and stress conditions (Zechmann 2017, 2020). Although it seems surprising at first that chloroplasts of non-stressed plants have comparatively low glutathione levels, their ability to synthesize glutathione rapidly upon high light stress, such as within 90 s when *Arabidopsis* plants were transferred from 50 to 1000 µmol m^−2^ s^−1^ (Choudhury et al. 2018), seems to be sufficient for redox-homeostasis in this compartment and even to supply glutathione for redox-balancing of the cytosol (Maughan et al. 2010).

A high glutathione concentration in the nucleus protects nucleic acids, proteins and lipids from oxidation. Nuclear glutathione further plays a role in redox adjustments during the cell cycle (Diaz Vivancos et al. 2010b, 2010a). Redox-homeostasis in the nucleus is maintained by GSH and GSSG exchange with the cytosol via the nuclear pores and by nuclear GR activity (Dorion et al. 2021; Müller-Schüssele et al. 2021). The increased GSH demand of the nucleus, triggered by either salicylic acid or excess light treatment, upregulated glutathione synthesis at the transcriptional level in wheat and *Arabidopsis* (Diaz Vivancos et al. 2010a; Gasperl et al. 2021). For redox adjustments to excess light, a high nuclear glutathione demand seems to be important, whereas a relocation of glutathione to peroxisomes seems to facilitate adaptations to far-red light under Asc deficiency (Heyneke et al. 2013; Gasperl et al. 2021). Peroxisomes require glutathione mainly to counteract H_2_O_2_ from SOD- or glycolate oxidase-associated reactions (Noctor et al. 2018). As mentioned above, glutathione in peroxisomes of stressed plants scavenges excess H_2_O_2_ from photorespiration under excess light or osmotic stress (Zechmann 2014; Gasperl et al. 2021).

Studies using *Arabidopsis* roGFP reporter lines that targeted specific cell compartments showed that in most of these compartments, glutathione is maintained in a reduced redox state under non-stress conditions. Exceptions are the ER lumen, the vacuole and the apoplast, where GR, to regenerate GSH from GSSG, is absent (Noctor et al. 2012; Müller-Schüssele et al. 2021). A more positive reduction potential (E_*h*_) and evidence for the presence of GT activity, which may use GSSG in the vacuole to reduce organic hydroperoxides (Öztetik 2008), was recently detected in vacuoles of dormant red beet taproot cells (Pradedova et al. 2019). The distinct glutathione (and Asc) level and its redox state in individual cell compartments allow control of the local subcellular redox environment and gradient, which affects many redox-dependent metabolic processes and is therefore considered a valuable marker for the plant stress response (Foyer and Noctor 2009; Noctor et al. 2012, 2013; Müller et al. 2014; Zechmann 2020; Dorion et al. 2021).

## Modifications in compartment-specific glutathione distribution and redox-state by light intensity and spectrum composition

Subcellular total glutathione levels changed with the diurnal rhythm in *Arabidopsis* (except for vacuoles), reaching a maximum after 3 h of light (150 µmol m^−2^ s^−1^), followed by a strong decrease within the next 1–2 h and a minimum at the end of the night. A much lower glutathione concentration seems to result from limited availability of the glutathione precursors glycine (from reduced photorespiration) and cysteine (from reduced sulfur uptake and incorporation) during darkness (Buwalda et al. 1990; Noctor et al. 1997, 1999; Huseby et al. 2013). The depleted glutathione level was restored when plants were exposed to light (150 µmol m^−2^ s^−1^) and fed with glycine or cysteine, respectively (Zechmann et al. 2007, 2008b; Höller et al. 2010; Király et al. 2012). Accordingly, excess light induced cysteine supply, glutathione synthesis (chloroplastic and more prominently cytosolic *GSHS*) and reduction (cytosolic *GR*) in wheat (Gasperl et al. 2021). A gradual increase in the chl-roGFP oxidation state (chl-*E*_*GSH*_) was recently reported in a chloroplast-targeted redox-sensitive (chl-roGFP2) potato reporter line to positively correlate with gradually increasing light intensity from 200 up to 720 and 1250 µmol m^−2^ s^−1^, respectively. The oxidizing effect was reversible by returning to a light intensity of 200 µmol m^−2^ s^−1^ after 14 h. A particularly strong effect on the potato chl-*E*_*GSH*_ in older leaves was found when the higher light intensities were combined with cold treatment (3 °C). Increased chl-*E*_*GSH*_ did not recover when the light intensity was lowered to 200 µmol m^−2^ s^−1^ after 10 h. The effect was, however, dependent on the developmental stage of the leaves, as newly formed upper leaves showed no increase in chl-roGFP oxidation state. This discrepancy may be explained by the comparatively low photosynthetic capacity of young leaves and by the induction of photo-protective mechanisms (non-photochemical quenching, photorespiration) (Hipsch et al. 2021). In accordance with the previous findings, organelle-specific responses to changes in light intensity were detected by redox-sensitive *Arabidopsis* reporter lines (roGFP2). The redox state shifted towards oxidation in peroxisomes, organelles associated with the detoxification of ROS and H_2_O_2_ from photorespiration, during 22 h of darkness, whereas in chloroplasts, which are organelles associated with glutathione synthesis, the redox state was specifically affected by short-term excess light (3 h at 600 µmol m^−2^ s^−1^) (Bratt et al. 2016). Haber and Rosenwasser (2020) reported immediate oxidation of *Arabidopsis* chl-roGFP after transition from darkness to light, which was more severe at light intensities of 750–1700 µmol m^−2^ s^−1^ than at 220–650 µmol m^−2^ s^−1^, and followed by gradual reduction of the chl-roGFP, which impressively demonstrates the versatility of the plant subcellular redox environment in response to changes in light.

Low light intensity (50 µmol m^−2^ s^−1^) for a few hours (4 h) did not alter the total glutathione level in the organelles of *Arabidopsis* wildtype plants. However, it was lower compared than that at the control light intensity (150 µmol m^−2^ s^−1^) in peroxisomes of the Asc-deficient *Arabidopsis vtc2-1* mutant (Heyneke et al. 2013). Similarly, in peroxisomes, nuclei and the cytosol of wheat cultivated at the same low light intensity for several days, decreased total glutathione levels were detected compared to plants grown at 250 µmol m^−2^ s^−1^ (Gasperl et al. 2021). *Arabidopsis vtc2-1* accumulates only 10 to 30% of the wildtype Asc level, which results from a mutation in the GDP-l-galactose phosphorylase 1 gene, the enzyme catalyzing an intermediate step in Asc synthesis (Müller-Moulé et al. 2004; Linster et al. 2007). Excess light (300, 700, 1500 µmol m^−2^ s^−1^) for a few hours stimulated total glutathione accumulation in the *Arabidopsis* wildtype, particularly in organelles associated with glutathione synthesis (chloroplasts and the cytosol) and detoxification of ROS and H_2_O_2_ from photorespiration (peroxisomes), whereas in the glutathione-deficient *Arabidopsis pad2-1* mutant, total glutathione increased in mitochondria, where ROS are frequently formed during respiration (Heyneke et al. 2013). *Arabidopsis pad2-1* accumulates only approximately 20% of the wildtype glutathione level (except for mitochondria), which results from a mutation in the γ-ECS (GSH1) gene, the enzyme catalyzing the rate-limiting step in glutathione synthesis (Parisy et al. 2007; Zechmann and Müller 2010; Koffler et al. 2011). *Arabidopsis* wildtype, the *pad2-1* and *vtc2-1* mutants and the moderately frost-sensitive wheat variety Chinese Spring adapted to excess light at 500 µmol m^−2^ s^−1^ (control light intensity: 250 µmol m^−2^ s^−1^) for several days by increasing glutathione concentrations. At the subcellular level, excess light stimulated total glutathione accumulation, particularly in chloroplasts, the cytosol and peroxisomes, in the *Arabidopsis pad2-1* mutant and wheat (Gasperl et al. 2021).

Long-term excess light at 300 and 700 µmol m^−2^ s^−1^ for 2 weeks enhanced total glutathione (and Asc) accumulation in the stroma of *Arabidopsis* wildtype chloroplasts. Only plants grown at 1500 µmol m^−2^ s^−1^ accumulated glutathione (and Asc) in the stroma and inside the thylakoid lumen. *Arabidopsis* wildtype chloroplasts were able to counteract oxidative stress from 14 days of exposure to excess light at up to 1500 µmol m^−2^ s^−1^ in the mesophyll of the leaf center, as H_2_O_2_ concentration did not alter in this area (in contrast to short-term excess light; see above), and total glutathione (and Asc) level increased markedly; this local increase in antioxidants was accompanied by structural and ultrastructural adaptations, namely reduction of chloroplast number and thylakoids and accumulation of plastoglobuli, respectively (Heyneke et al. 2013). Plastoglobuli are in close contact with thylakoid membranes and serve as biosynthesis and storage subcompartments, for example for carotenoids and xanthophylls (Austin et al. 2006). Plastoglobuli accumulate during thylakoid membrane re-organization and degradation in response to stress and thus play an important role in the adjustment of chloroplasts to stress (Espinoza-Corral et al. 2021; Zechmann et al. 2021). Excess light for 5 days at 500 µmol m^−2^ s^−1^ (control light intensity: 120 µmol m^−2^ s^−1^) likewise led to plastoglobuli accumulation and thylakoid membrane degradation/re-organization and increased carotenoid content in plastoglobuli in *Arabidopsis* wildtype plants and induced proteins associated with leaf senescence and jasmonic acid biosynthesis (Espinoza-Corral et al. 2021). Specifically, additional adjustments to 14 days of exposure to excess light at 1500 µmol m^−2^ s^−1^ were found in the glutathione (*pad2-1*)- and Asc (*vtc2-1*)-deficient mutants of *Arabidopsis*. In comparison to the wildtype and *vtc2-1* mutant, the *pad2-1* mutant seems to compensate its glutathione deficiency by maintaining comparatively high levels of Asc in peroxisomes and by forming an additional layer of palisade cells (Heyneke et al. 2013). Thickened leaves as a response to high light intensity are also known from other plant species (Björkman 1981; Pearcy 1998). Total glutathione increased in most compartments of the *vtc2-1* mutant, but particularly in the chloroplasts and nuclei (Heyneke et al. 2013). The depletion of cytosolic glutathione by import into the nucleus induces glutathione synthesis, which accelerates the accumulation of GSH and allows the maintenance of a reducing redox environment under stress conditions (Diaz Vivancos et al. 2010a). It would appear that the *vtc2-*1 mutant seems to compensate its lack of Asc by a combination of glutathione re-location and synthesis. At the whole leaf level o thef *Arabidopsis* wildtype, increase in photo-protective anthocyanins was visible in plants grown at 300 µmol m^−2^ s^−1^, which was more pronounced, but also accompanied by small necrotic lesions at leaf edges at 700 µmol m^−2^ s^−1^, and intensified even more at 1500 µmol m^−2^ s^−1^, while the photosynthesis-associated pigments (chlorophyll and carotenoids) were reduced (Heyneke et al. 2013).

White light with a lower red/far-red ratio induces cysteine and glutathione metabolism in wheat, but not at the transcriptional level (Monostori et al. 2018; Toldi et al. 2019; Gasperl et al. 2021). Lower red/far-red ratio (blue/red 1:5; red/far-red: 10:1) at 250 µmol m^−2^ s^−1^ for several days raised GSH and GSSG concentrations compared to white light (blue/red 1:2; red/far-red: 15:1) of the same intensity; GSH increased, however, to a greater extent. Thus, the GSSG/GSH ratio was at the same level (~ 0.1) as in wheat leaves grown under white light of the same intensity. Leaves of the *Arabidopsis* wildtype and *vtc2-1* and *pad2-1* mutations maintained largely the same GSH and GSSG concentrations and GSSG/GSH ratio (0.1) when grown under lower red/far-red ratio or white light spectrum, respectively. Total glutathione labeling density in the *Arabidopsis* wildtype and the *vtc2-1* mutant, except for peroxisomes (1.8-fold increase) in the *vtc2-1* mutant, was likewise largely unaffected by the decreased red/far-red ratio. In the *Arabidopsis* *pad2-1* mutant, total glutathione labeling density increased in nuclei by twofold, and in peroxisomes and the cytosol by onefold; in wheat, it decreased in cytosol by 1.8-fold, in chloroplasts by 1.5-fold and in nuclei and peroxisomes, by onefold respectively, in comparison to white light at the same intensity. Similar to excess light response, cysteine, GSSG and GSH in wheat leaf extracts increased under the lower red/far-red ratio. However, in contrast to excess light, GSH supply either from de novo synthesis and/or reduction was not transcriptionally upregulated (Gasperl et al. 2021). For this experimental setting and from work involving even higher light intensities, we can therefore conclude that *Arabidopsis* and wheat plants are able to adjust their subcellular redox environment to different light intensities (Heyneke et al. 2013; Gasperl et al. 2021) and to a lower red/far-red ratio, and that the adaptations in subcellular glutathione distribution are similar under excess light and a lower red/far-red ratio in the *Arabidopsis pad2-1* mutant and wheat (Gasperl et al. 2021).

## Conclusion

The increase in ROS and H_2_O_2_ along the photosynthetic electron transport chain in chloroplast thylakoids and the subsequent transfer of excess ROS and H_2_O_2_ from photorespiration in peroxisomes into the cytosol and vacuoles under excess light initiates the accumulation and redistribution of antioxidants (glutathione, ascorbate) in plant cell compartments (Fig. 1). Under a lower red/far-red ratio, we propose that an increase in ROS and H_2_O_2_ in chloroplasts arises from inefficient chlorophyll biosynthesis in seedlings after transition to white light or in mature shaded leaves from an imbalance in the excitation state of the two photosystems (short term) and limited availability of reductants and assimilates (long term), which again initiates the accumulation and redistribution of glutathione (and ascorbate) in plant cell compartments (Fig. 1). Choosing the most suitable method for glutathione localization and quantification studies in subcellular compartments of plants depends on the cell layers (epidermis or mesophyll, vascular tissue), cell compartments and form of glutathione (total glutathione or redox state of glutathione) that studies target (Table 1). Limiting the auto-oxidation and/or redistribution of glutathione during analyses is generally crucial for obtaining reliable results. Light intensity-mediated redox shifts in plant cells are compartment specific, time dependent and reversible under non-stress conditions. Light intensity signaling by phyA and possibly by ROS into the nucleus induces glutathione synthesis and reduction at the transcriptional level in wheat. Differences between *Arabidopsis* and wheat in response to moderate excess light regimes at the subcellular level may arise from the different light intensity optima (*Arabidopsis*: 100–150 µmol m^−2^ s^−1^, wheat: 250 µmol m^−2^ s^−1^) of both plants, and *Arabidopsis* may require thus faster signaling and adaptation. Differences between *Arabidopsis* and wheat in terms of their response to a lower red/far-red ratio at the subcellular level may arise, because in *Arabidopsis*, leaves of lower nodes are partially shaded by younger leaves of the same plant, while wheat is grown in dense rows and leaves of lower nodes are shaded by younger leaves of the same plant and by neighboring plants.

Improved drought tolerance in a wheat variety and in soybean after excess light or shade pre-treatment is at least partly linked to antioxidants and associated enzyme activities. Moderate excess light or far-red light may in the future allow the priming of crop seedlings against certain stress conditions prior to transplanting them into the field.

## Abbreviations

^1^O_2_: Singlet oxygen
ADP: Adenosine diphosphate
AOX: Alternative oxidase
APX: Ascorbate peroxidase
Asc: Ascorbate
ATP: Adenosine triphosphate
*cat*: Catalase knockout
CAT: Catalase
chl-*E*_*GSH*_: Chl-roGFP oxidation state
chl-roGFP2: Chloroplast targeted roGFP
CLSM: Confocal-laser scanning microscopy
cry: Cryptochrome
DTT: Dithiotreitol
E_*h*_: Reduction potential
ER: Endoplasmic reticulum
FIN219: Far-Red Insensitive 219
FIP1: FIN219-interacting protein 1
GGT-1: Gamma-glutamyl transferase/transpeptidase 1
*ggt*: GGT-1 knockout
GR: Glutathione reductase
GSH: Reduced glutathione
GSHS: Glutathione synthetase
GSSG: Oxidized glutathione (= glutathione disulfide)
GSSG/GSH: Reduction state of glutathione
GT: Glutathione transferase
H_2_O_2_: Hydrogen peroxide
HPLC: High performance liquid chromatography
HyPer2: Fluorescent H_2_O_2_ biosensor
IgG: Immunoglobulin G
immunolabeling: Total glutathione localization by use of a specific antibody
LEDs: Light-emitting diodes
mbrB: Monobromobimane
mchlB: Monochlorobimane
NADP^+^: Nicotinamide adenine dinucleotide phosphate
NADPH: Reduced form of NADP^+^
NEM: N-ethylmaleimide
O_2_^−^: Superoxide radicals
*pad*: Phytoalexin deficient
phyA: Phytochrome A
PS II: Photosystem II
roGFP: Redox-sensitive green fluorescence protein
ROS: Reactive oxygen species
SOD: Superoxide dismutase
TEM: Transmission electron microscope
Total glutathione: Sum of reduced (GSH) and oxidized (GSSG) glutathione, detected via immuno-labeling
*vtc*: Vitamin C1 gene
γ-EC: γ-Glutamylcysteine
γ-ECS: γ-Glutamylcysteine synthetase
